# Elevation of brain-enriched miRNAs in cerebrospinal fluid of patients with acute ischemic stroke

**DOI:** 10.1186/s40364-017-0104-9

**Published:** 2017-07-11

**Authors:** Sofie Sølvsten Sørensen, Ann-Britt Nygaard, Anting Liu Carlsen, Niels H. H. Heegaard, Mads Bak, Thomas Christensen

**Affiliations:** 1 Department of Neurology, Nordsjællands Hospital, University of Copenhagen, Dyrehavevej 29, 3400 Hillerød, Denmark; 2 Department of Clinical Biochemistry, Nordsjællands Hospital, Hillerød, Denmark; 3 Department of Autoimmunology and Biomarkers, Statens Serum Institut, Copenhagen, Denmark; 4 Department of Cellular and Molecular Medicine, Panum Institute, University of Copenhagen, Copenhagen, Denmark

**Keywords:** Stroke, MicroRNA, Biomarkers, Brain ischemia, Cerebrospinal fluid

## Abstract

**Background:**

The purpose of this study was to investigate the potential of cerebrospinal fluid miRNAs as diagnostic biomarkers of acute ischemic stroke using three different profiling techniques in order to identify and bypass any influence from technical variation.

**Methods:**

Cerebrospinal fluid (CSF) from patients with acute ischemic stroke (*n* = 21) and controls (*n* = 21) was collected by lumbar puncture. miRNA analysis was performed with three different methods: 1) Trizol RNA extraction followed by Illumina Next Generation Sequencing (NGS) on all small RNAs, 2) Exiqon RNA extraction protocol and miRNA qPCR assays, and 3) validation of 24 selected miRNAs with Norgen Biotek RNA extraction protocol and Applied Biosystems qPCR assays.

**Results:**

NGS detected 71 frequently expressed miRNAs in CSF of which brain-enriched miR-9-5p and miR-128-3p were significantly higher in CSF of stroke patients compared to controls. When dividing stroke patients into groups according to infarct size several brain-enriched miRNAs (miR-9-5p, miR-9-3p, miR-124-3p, and miR-128-3p) were elevated in patients with infarcts >2 cm3. This trend appeared in data from both NGS, qPCR (Exiqon), and qPCR (Applied Biosystems) but was only statistically significant in some of the measurement platforms.

**Conclusions:**

Several brain-enriched miRNAs are elevated in the CSF three days after stroke onset, suggesting that these miRNAs reflect the brain damage caused by ischemia. The expression differences seem, however, limited to patients with larger ischemic brain injury, which argues against the use of CSF miRNAs as diagnostic biomarkers of stroke based on current methods.

**Electronic supplementary material:**

The online version of this article (doi:10.1186/s40364-017-0104-9) contains supplementary material, which is available to authorized users.

## Background

Ischemic stroke is a frequent cause of death and disability all over the world. In patients with atypical or discrete symptoms the diagnosis of ischemic stroke can be difficult and determining the cause of stroke in each individual is even more complicated. Despite thorough clinical and paraclinical evaluation comprising brain computed tomography (CT) or magnetic resonance imaging (MRI), blood tests, cardiac workup, and ultrasound examination of the carotid arteries, the cause of acute ischemic stroke remains unknown in 25% of all patients [1]. A biomarker with the ability to distinguish between different stroke etiologies (large-artery atherosclerosis, small-artery occlusion, and cardio-embolism) could potentially aid the physicians in choosing the best secondary prevention for their patients since treatment of the different stroke subtypes is not the same. In patients with large-artery atherosclerosis or small artery occlusion, anti-platelet drugs are first choice treatment for reducing the risk of stroke recurrence [2]. In contrast, in patients with cardioembolic stroke, anticoagulant therapy is the most effective prevention [3, 4]. Surgical intervention with carotid endarterectomy is considered in large-artery atherosclerosis as it reduces recurrent strokes in patients with a significant ipsilateral carotid artery stenosis [5]. The current and most widely used system for categorization of ischemic stroke subtypes, the TOAST classification, is based on clinical and paraclinical test results and has only moderate inter-rater reliability [6, 7]. Furthermore, many stroke patients have competing possible causes, such as concurrent atrial fibrillation and carotid artery stenosis, making it impossible to establish a specific etiology. Therefore, new diagnostic and prognostic biomarkers in relation to ischemic stroke are warranted and could potentially benefit stroke patients in various ways.

MicroRNAs (miRNAs) are small non-coding RNA molecules involved in numerous physiological processes in which they function as posttranscriptional inhibitors of gene expression. Their presence in different body fluids such as blood and CSF combined with their stability during long term storage make them interesting as biomarker candidates [8–10]. Recently, several clinical stroke studies have suggested a diagnostic value of miRNAs extracted from plasma/serum and circulating blood cells [11–15], although the results points in various directions. Since CSF is separated from the blood by the blood brain barrier (BBB) and because of its proximity to the brain tissue, it is obvious to assume that the CSF could contain unique signatures of miRNA expression specific for various central nervous system (CNS) pathologies, and therefore serve as a more valid biomarker source compared to other body fluids.

Within few minutes after stroke onset, various brain functions begin to break down as a direct consequence of ischemia including changes in gene expression [16, 17]. Microarray studies have identified several gene families, e.g. immediate early genes, heat shock protein genes, and genes related to apoptosis and inflammation that are regulated by focal brain ischemia [18–20]. Several experimental studies have shown changes in the expression of miRNAs in rat brain tissue following middle cerebral artery occlusion [21–26]. Based on this knowledge, we hypothesized that the changes in miRNA expression in the brain of acute ischemic stroke patients are reflected in the cerebrospinal fluid (CSF) surrounding the central nervous system.

Previously, we have explored the expression of miRNAs in CSF of acute stroke patients in a pilot study [27]. We found 21 frequently expressed miRNAs in the CSF of which two miRNAs (miR-221-3p and let-7c-5p) appeared to be up-regulated in stroke patients compared to controls, although not statistically significant after correction for multiple testing.

The purpose of this follow-up study was 1) to investigate the potential of CSF miRNAs as biomarkers of acute ischemic stroke using three different profiling techniques in order to identify and bypass any influence from technical variation and 2) to validate our previous findings in a larger group of patients.

## Methods

### Patient material and sample collection

Patients admitted with acute ischemic stroke at the Department of Neurology, Nordsjællands Hospital, University of Copenhagen, from June 2013 to December 2014 were asked to participate in accordance with the approval from the Danish Research Ethics Committee (project ID: H-I-2011-094).

As in our previous study, the stroke diagnoses were based on patient history, thorough neurologic examination and CT scan of the brain on admission. In addition, all patients were scanned on a 3 Tesla System Philips Achieva MRI scanner on the third day after symptom onset. The imaging protocol consisted of diffusion-weighted and T_2_-weighted sequences. Infarct volumes were calculated from MR diffusion-weighted images by multiplying the summed areas of infarction with the distance between each section [28]. After additional Doppler Ultrasound of carotid arteries and cardiac telemetry for at least 48 h during admission patients were categorized according to stroke subtype using the TOAST classification [6]. Controls were recruited from our outpatient neurology clinic as they underwent planned lumbar punctures as part of their investigation program for neurologic diseases others than stroke. A total of 42 patients were included, *n* = 21 ischemic stroke patients, and *n* = 21 controls. Patient characteristics are given in Table 1. A more detailed description of the different diagnoses and stroke parameters in each individual patient is given in Additional file 1. The composition of diagnoses and severity of stroke symptoms were comparable to our previous study, Additional file 2.

**Table 1:** Patient characteristics

Characteristics	Stroke (*n* = 21) Mean (range) or n (%)	Control (*n* = 21) Mean (range) or n (%)
Age (years)	66.6 (47–78)	66.0 (31–86)
Male sex	12 (57.1)	13 (61.9)
NIHSS	2.5 (0–10)	-
Infarct (cm^3^)	6.7 (0.3–32.4)	-
SAO	11 (52.4)	-
LAA	5 (23.8)	-
CE	2 (9.5)	-
U	3 (14.3)	-
Dementia	-	7 (33.3)
MCI	-	2 (9.5)
Multiple sclerosis	-	1 (4.8)
ALS	-	1 (4.8)
No CNS disease	-	10 (47.6)

*NIHSS* National Institute of Health Stroke Scale, *SAO* stroke caused by small-artery occlusion *LAA*, stroke caused by large-artery atherosclerosis, *CE* stroke caused by cardioembolism, *U* stroke of undetermined etiology,*MCI* mild cognitive impairment, *ALS* amyotrophic lateral sclerosis, *No CNS disease* patients with no evidence of disease in the central nervous system (benign headache, Bell’s palsy, polyneuropathy, restless legs or lower back pain)

Lumbar punctures were done by sterile technique on the third day after stroke onset with the use of a 0.7 mm spinal needle in accordance with local guidelines. At least 5 ml CSF were collected from each patient. For miRNA detection 1 ml CSF from each patient was centrifuged at 2000 x g at 4 °C for 15 min immediately after collection and the cell-free fractions were stored at −80.0 °C. Analyses of miRNAs were performed by three different methods as described below. A detailed description of the methodology is available online, Additional file 3.

### Trizol RNA extraction followed by Illumina next generation sequencing

RNA was isolated from 100 μl CSF using TriZol reagent (Invitrogen) according to the manufacturer’s protocol. Small RNA libraries were prepared using the TruSeq small RNA library preparation kit (Illumina). The resulting cDNA was amplified by PCR and all small RNA sequences were purified on gels and sequenced on a NextSeq500 (Illumina). The libraries from two stroke patient samples were of low quality and were therefore excluded from further analysis. Reads were trimmed for adapters and reads shorter than 15 nucleotides in length were discarded. Small RNAs were aligned to the human genome (hg19) and miRbase (v20) using sRNAbench [29]. miRNA counts were normalized in R Bioconductor package edger [30] using Trimmed Mean of M-values (TMM). Fold changes were based on the average count in each patient group.

### Exiqon RNA extraction protocol and miRNA qPCR assays

RNA extractions and sample preparations were done by Exiqon Services, Denmark. Total RNA was extracted from 200 μl CSF using spin column chromatography (miRCURY™ RNA isolation kit for bio fluids). RNA was reverse transcribed using the miRCURY LNA™ Universal RT miRNA kit (Exiqon). Each miRNA was assayed once by qPCR on the miRNA Ready-to-Use PCR, Human panel I containing 372 specific miRNA primers. Amplification was performed in a LightCycler® 480 Real-Time PCR System (Roche) in 384 well plates. Using NormFinder [31] the best normalizer was identified as the average Ct value of the 9 miRNAs detected in all samples (miR-15a-5p, miR-21-5p, miR-23a-3p, miR-23b-3p, miR-99a-5p, miR-125b-5p, miR-145-5p, miR-204-5p, and miR-320a) and relative expression levels (RELs) were calculated by the formula *REL* = 2^*miR average* − *miR*^/2^*Global mean average* − *Global mean*^ based on the principle of 2^-∆∆Ct^ [32]. Fold changes were based on the median value of REL in each patient group (missing values excluded).

### Norgen Biotek RNA extraction protocol and applied biosystems qPCR assays

Total RNA was extracted from 200 μl CSF using spin column chromatography (Total RNA Purification Kit, Norgen Biotek). Reverse transcription was performed by TaqMan microRNA Reverse Transcription Kit performed on a 2720 Thermal Cycler (Applied Biosystems). miRNA specific pre-amplification was accomplished using TaqMan PreAmp master mix and TaqMan MicroRNA Assays (Applied Biosystems). Pre-amplified samples and TaqMan miRNA assays for 24 selected miRNAs were applied to a 48.48 Dynamic Array IFC chip and amplified on a BioMark real-time microfluidic PCR system (Fluidigm).

Normfinder [31] identified two equally good normalizers: miR-320a and the average Ct value of the 20 miRNAs detected in all samples. Based on the assumption that most of the 24 miRNAs in this validation experiment were differentially expressed between our patient groups we wanted to avoid a global mean normalization that could potentially undermine any differences by comparing raw data to the average of all miRNAs. Therefore, miR-320a was chosen as normalizer and RELs were calculated using the formula *REL* = 2^*miR average* − *miR*^/2^*miR*−320*a average* − *miR*−320*a*^ based on the principle of 2^-∆∆Ct^ [32]. Fold changes were based on the median value of REL in each patient group (missing values excluded).

### Statistics

Two-sided nonparametric Mann-Whitney tests were performed for REL comparisons between groups (SPSS software). In the NGS experiment differentially expressed miRNAs were detected by an exact test based on conditional maximum likelihood (CML) included in the R Bioconductor package edgeR [30]. *P* values from NGS and qPCR were corrected for multiple testing with the Benjamini-Hochberg (BH) procedure and *p* < 0.05 were considered significant. In all group comparisons missing expression values were treated as zero. The NGS heat map was generated using Multiple Experiment Viewer [33]. Selection of the best miRNA predictors was based on logistic lasso regression [34] performed on the top 15 most deregulated miRNAs detected by NGS. AUC and classification accuracy was estimated by leave-one-out cross-validation based on the seven predictors selected by the lasso. Differences in total numbers of miRNAs between groups as well as differences in age were analyzed by two-sided parametric t tests. Fischer’s exact test was used for analysis of sex differences.

## Results

### Overall detection by NGS and Exiqon qPCR (human panel I)

A total of 246 miRNAs were detected in the CSF by NGS of which 71 miRNAs were expressed in more than 90% of the patient samples. Slightly fewer, 210 miRNAs, were detected by the qPCR screening (Exiqon, human panel I) of which only 21 miRNAs were expressed in more than 90% of the samples. With the exception of miR-1246 all of the differentially expressed miRNAs between stroke patients and controls were detected by both NGS and qPCR (Additional file 4) although the detection rate was generally lower in the qPCR experiment.

### Results from NGS

**Fig. 1:**
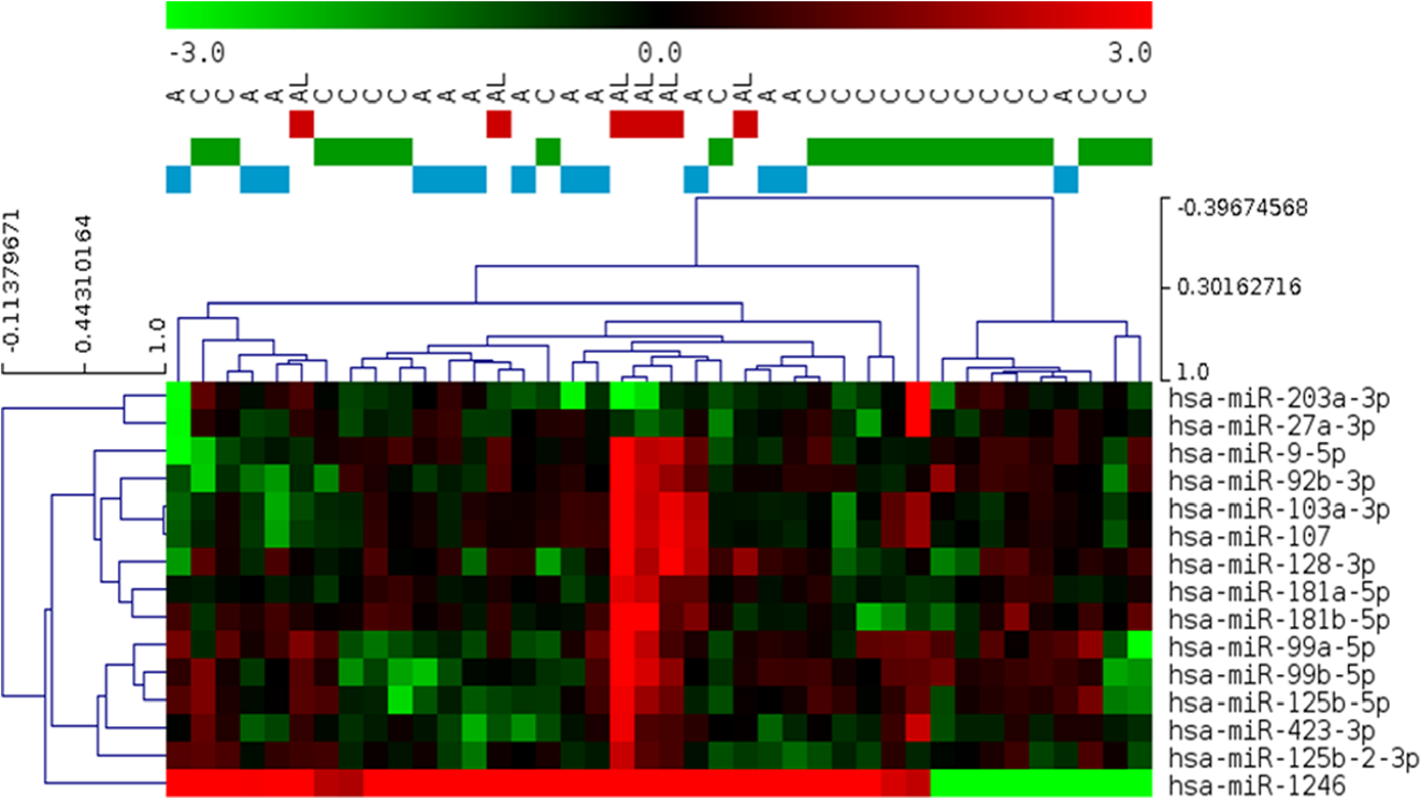
Heatmap showing logFC expression of the top 15 most differentially expressed miRNAs between stroke patients and controls detected by Next Generation Sequencing. Fold changes were calculated as the expression in each sample (normalized count) relative to the average expression in the control group. *A*: stroke patients with infarcts <2 cm^3^; *AL*: stroke patients with infarcts >2 cm^3^; *C*: control patients

In the NGS experiment only two miRNAs (miR-1246 and miR-128-3p) were significantly up-regulated in the stroke group compared to controls after BH correction. The up-regulation of miRNAs was most evident in patients with large-artery atherosclerosis or cardioembolism whose infarcts were larger (mean volume (range) = 16.5 cm^3^ (2.5–32.4)) compared to patients with small-artery occlusion (mean volume (range) = 0.7 cm^3^ (0.3–1.6)) (Fig. 1). When dividing patients into groups according to infarct size 8 miRNAs (miR-9-5p, miR-99b-5p, miR-103a-3p, miR-107, miR-128-3p, miR-181a-5p, miR-181b-5p, and miR-1246) were significantly up-regulated in patients with infarcts >2.5 cm^3^ (*n* = 7) compared to stroke patients with smaller infarcts (*n* = 12).

### Results from qPCR screening (Exiqon, human panel I)

Consistent with our previous findings [27] qPCR (human panel I, Exiqon) detected only 21 frequently expressed miRNAs in CSF of which none were differentially regulated between stroke patients and controls after BH correction. The up-regulation of miR-221-3p and let-7c-5p found in our previous pilot study could not be validated. By considering the expression in subgroups with different infarct sizes two miRNAs (miR-9-3p and miR-124-3p) were significantly up-regulated in stroke patients with infarcts >2 cm^3^ (*n* = 8) compared to stroke patients with smaller infarcts (*n* = 13).

### Validation of selected miRNAs by qPCR (applied biosystems)

**Fig. 2:**
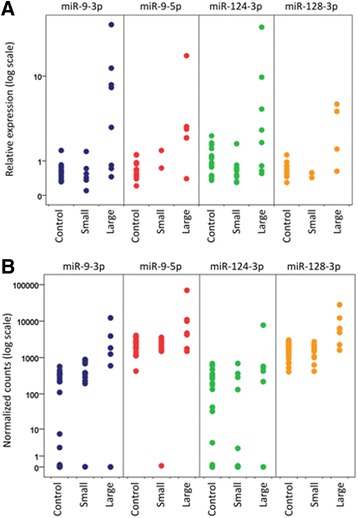
Scatter plots of the expression levels of four brain-enriched miRNAs (miR-9-3p, miR-9-5p, miR-124-3p, and miR-128-3p) detected by Next Generation Sequencing (A) and Exiqon qPCR (B). *Large*: stroke patients with infarcts >2 cm^3^ (*n* = 13); *Small*: stroke patients with infarcts <2 cm^3^ (*n* = 8); *Control*: control patients with various neurological diseases other than stroke (*n* = 21)

Following the completion of NGS and the first qPCR experiment we selected miRNAs that showed differential expression between stroke patients and controls (and/or between patients with small and large infarcts) for technical validation. The selection was based on significant unadjusted *p* values (*p* < 0.05) and/or a large fold change (0.3 > FC > 3) in at least one of the two experiments. A total of 21 selected miRNAs were included in the validation study (normalizers excluded). Table 2 summarizes results for the 21 selected miRNAs measured with NGS, qPCR (Exiqon), and qPCR (Applied Biosystems), respectively. Five miRNAs (miR-9-5p, miR-9-3p, miR-107, miR-124-3p, and miR-128-3p) were consistently up-regulated in the group of patients with infarcts >2 cm^3^, although not statistically significant throughout all measurement platforms. At least four of them (miR-9-5p, miR-9-3p, miR-124-3p, and miR-128-3p) are known as brain-enriched miRNAs with a high tissue specificity index [35]. miRNA-1246 showed the highest up-regulation by NGS (FC = 13.6; *p* = 0.01) but did not appear to be up-regulated by qPCR (Applied Biosystems) and was unfortunately not included in the human panel I (Exiqon). Fig. 2 illustrates the expression levels of the four brain-enriched miRNAs in different patient subgroups.

**Table 2:** Expression of 21 selected miRNAs detected by three different methods

miRNA	NGS			Exiqon qPCR			Applied Biosystems qPCR		
	Detection(%)	Stroke/Control FC (*P*)	Large/Small FC (*P*)	Detection(%)	Stroke/Control FC (*P*)	Large/Small FC (*P*)	Detection(%)	Stroke/Control FC (*P*)	Large/Small FC (*P*)
let-7b-5p	95	0.8 (0.99)	2.3 (0.73)	98	1.1 (0.99)	0.6 (0.29)	100	1.0 (0.89)	0.6 (0.57)
let-7c-5p	100	0.7 (0.99)	0.9 (0.99)	74	0.7 (0.99)	0.6 (0.11)	95	0.7 (0.71)	0.3 (0.88)
miR-9-5p	98	2.8 (0.06)	7.0 (0.00)	48	3.3 (0.90)	2.3 (0.07)	100	0.9 (0.95)	10.0 (0.01)
miR-9-3p	70	7.1 (0.08)	6.8 (0.28)	70	1.4 (0.85)	9.8 (0.01)	100	1.2 (0.73)	1.9 (0.66)
miR-15a-5p	63	1.0 (0.99)	2.0 (0.99)	100	1.0 (0.50)	2.2 (0.31)	100	1.0 (0.96)	2.4 (0.44)
miR-30d-5p	100	1.2 (0.99)	1.3 (0.79)	91	1.6 (0.45)	1.1 (0.48)	100	1.7 (0.01)	6.2 (0.09)
miR-99b-5p	100	1.9 (0.32)	4.2 (0.00)	38	0.7 (0.99)	0.9 (0.84)	72	0.4 (0.43)	0.5 (0.70)
miR-103a-3p	100	1.9 (0.17)	4.3 (0.00)	48	1.0 (0.84)	0.4 (0.80)	100	0.6 (0.49)	0.9 (0.85)
miR-107	100	2.0 (0.13)	4.5 (0.00)	31	0.6 (0.71)	n.d. small	100	3.6 (0.37)	3.5 (0.58)
miR-124-3p	58	2.7 (0.99)	11.0 (0.19)	83	0.9 (0.99)	3.6 (0.03)	100	1.1 (0.92)	1.7 (0.74)
miR-128-3p	100	2.5 (0.04)	5.5 (0.00)	41	1.0 (0.95)	2.7 (0.15)	100	0.6 (0.42)	1.5 (0.88)
miR-129-5p	60	2.6 (0.99)	2.3 (0.99)	26	1.1 (0.91)	3.1 (0.14)	100	1.5 (0.97)	1.0 (0.77)
miR-181a-5p	100	1.5 (0.40)	3.0 (0.00)	33	0.9 (0.78)	0.6 (0.49)	100	0.5 (0.33)	0.7 (0.76)
miR-181b-5p	100	1.8 (0.32)	3.1 (0.02)	5	n.d. control	n.d. small	100	0.9 (0.46)	0.7 (0.58)
miR-203a-3p	100	0.3 (0.02)	0.8 (0.99)	-	-	-	100	0.7 (0.85)	1.4 (0.78)
miR-204-5p	100	0.8 (0.99)	0.6 (0.73)	100	0.8 (0.90)	0.6 (0.13)	100	0.8 (0.46)	0.7 (0.06)
miR-210-3p	38	1.3 (0.99)	2.3 (0.99)	38	1.1 (0.99)	0.4 (0.06)	100	0.9 (0.52)	1.0 (0.81)
miR-221-3p	95	1.0 (0.99)	3.0 (0.63)	79	1.1 (0.97)	0.3 (0.33)	81	0.7 (0.32)	0.4 (0.52)
miR-485-5p	72	2.4 (0.99)	5.5 (0.46)	-	-	-	100	0.9 (0.54)	2.2 (0.73)
miR-497-5p	28	2.9 (0.99)	2.6 (0.99)	74	1.0 (0.94)	4.1 (0.32)	79	1.6 (0.44)	3.9 (0.65)
miR-1246	78	11.5 (0.02)	13.6 (0.01)	-	-	-	100	1.2 (0.40)	1.0 (0.80)

*Detection (%)*: percentage out of 42 samples in which the miRNA was detected; *Stroke/Control FC*: fold change of median miRNA expression levels in the stroke group (*n* = 21) compared to the control group (*n* = 21); *Large/Small FC*: fold change of median miRNA expression levels in a stroke subgroup with infarcts >2 cm^3^ (*n* = 8) compared to all other stroke patients (*n* = 13); *n.d.*: not detected. All *P* values were adjusted for multiple testing with the Benjamini-Hochberg procedure

### Classification accuracy and ROC curve

**Fig. 3:**
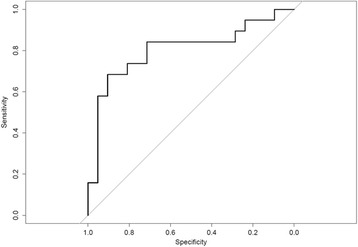
Receiver operating characteristic (ROC) curve showing the diagnostic value of the combination of 7 miRNAs selected by logistic lasso regression to discriminate stroke patients from controls. AUC = 0.80, classification accuracy = 0.75. miRNAs included in the model: miR-9-3p, miR-92b-3p, miR-99a-5p, miR-128-3p, miR-181a-5p, miR-203a-3p, and miR-1246

Although the strongest miRNA changes were seen in patients with larger infarcts we attempted to analyze the predictive power of one or more miRNAs to separate stroke patients from controls. Logistic lasso regression performed on the top 15 most differentially expressed miRNAs detected by NGS identified a combination of 7 miRNAs that could discriminate stroke patients from controls with a classification accuracy of 0.75. Fig. 3 shows the receiver operating characteristic (ROC) curve estimated by leave-one-out cross validation (AUC = 0.80).

## Discussion

Based on the hypothesis that acute stroke causes transcriptomic changes in the brain tissue which is reflected in the molecular profile of miRNAs in the CSF, we aimed to search for a miRNA expression profile that would uniquely identify stroke patients from other neurological patients. Such a marker could potentially be useful in situations where the diagnosis may be uncertain (e.g. transient ischemic attacks) or when the underlying cause of ischemic stroke remains unknown.

Obtaining a blood sample for measurement of miRNA biomarkers is less invasive than a lumbar puncture. However, many organs are likely to contribute to the extracellular miRNA content in plasma. Blood cells, in particular, have been shown to be major sources of plasma miRNAs. Pritchard et al. [36] showed that 58% of plasma miRNAs, reported in the literature to be candidate biomarkers for various cancers, are highly expressed in blood cells. This raises the concern that many miRNAs reported as circulating biomarkers reflect a secondary effect of blood cells rather than a disease-specific effect. Since CSF is separated from the circulation by the BBB and because of the continuous interchange between CSF and interstitial fluid [37], it seems more likely that the composition of miRNAs in CSF reflects CNS pathology better than that of blood plasma. We therefore chose to examine miRNA expression in cell-free CSF samples.

In this comprehensive investigation of miRNAs in the CSF from 21 stroke patients and 21 controls, encompassing both NGS and two different qPCR protocols, we identified relatively few frequently expressed miRNAs in the CSF. In general, the expressional variations within groups and small fold changes between groups resulted in no statistically differences for most of the detected miRNAs. However, it is noteworthy that brain-enriched miR-9-5p, miR-9-3p, miR-124-3p, and miR-128-3p were consistently up-regulated in the group of patients with infarcts >2 cm^3^ throughout all experiments.

Studies investigating the abundance of miRNAs in different human organs have identified miR-9-3p, miR-9-5p, miR-124-3p, and miR-128-3p as being enriched in various brain regions (midbrain, cortex, cerebellum and hippocampus) as well as being brain-specific with a high tissue specificity index [35, 38]. Cell studies suggest that miR-124 and miR-128 are predominantly expressed in neurons [39] and studies on embryonic stem cells imply that miR-9 and miR-124 play key roles in neural fate determination in mammalian brain development [40].

**Fig. 4:**
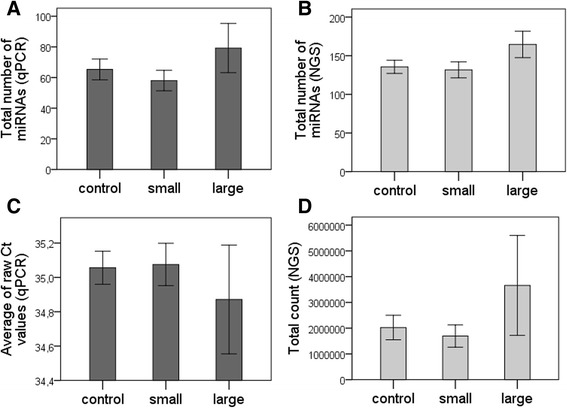
Total number of different miRNAs detected by qPCR (A) and NGS (B). The general amount of miRNA is expressed as the average of raw Ct values (C), and NGS total counts (D). All variables are shown as mean values (with standard error bars) among controls, patients with infarcts <2 cm^3^ (*small*), and patients with infarcts >2 cm^3^ (*large*)

It is unknown whether disease-associated miRNAs in CSF are involved in the pathogenic mechanisms of ischemic cell death in the CNS or reflect a passive molecular response to cerebral damage. Stroke-associated miRNAs in the CSF may originate from cytoplasmic protein complexes released during neuronal and glial cell lysis caused by brain ischemia. In this study, the overall amount of miRNA in the CSF seemed slightly higher in patients with larger infarcts compared to other stroke patients, although not statistically significant. The total number of different miRNAs detected by both qPCR and NGS was significantly higher in the group of patients with infarcts >2 cm^3^ (*p* = 0.03), which might indicate a general leak of miRNAs into the CSF following ischemic cell injury and ensuing cell lysis (Fig. 4).

Another possible origin of any miRNA in the CSF is active release of miRNAs enclosed in small membranous vesicles (exosomes) which may play a role in intercellular communication [41, 42]. Only one study has focused on exosome-derived miRNAs in stroke patients. Ji et al. [12] extracted exosomes from serum of 65 stroke patients and 66 non-stroke controls with matching age and cardiovascular risk factors. They found a generally higher concentration of exosomes and a significant increase of exosome derived miR-9 (FC = 16) and miR-124 (FC = 4) in the stroke group compared to controls, suggesting that exosomes from the brain can somehow cross the BBB and enter the peripheral circulation. Accordingly, Ogata et al. [43] have shown elevation of miR-9-3p in the hippocampus as well as in exosomes in peripheral blood of mice after administration of trimethyltin that selectively damages CNS neurons, indicating that miR-9-3p is a potential marker of brain damage.

If the elevation of specific miRNAs in the CSF of stroke patients is caused by expressional changes in the brain tissue, these changes could possibly both reflect the molecular chaos in the ischemic brain tissue as well as its counterregulatory mechanisms. To search for possible molecular functions we made a combined miRPath [44] (version 3.0) analysis of the up-regulated miR-9-3p, miR-9-5p, miR-107, miR-124-3p, and miR-128-3p (results merged by genes union, FDR correction, *P* < 0.05) which identified several KEGG pathways relevant to ischemic stroke, including pathways involved in p53 signaling, cell cycling, tumor necrosis factor (TNF) signaling, hypoxia inducible factor 1 (HIF-1) signaling and neurotrophin signaling.

In literature, the expression of miR-128 has been linked to alterations in the expression of genes implicated in apoptosis (Bcl-2, Bax, p53, and Bak) as well as angiogenesis (VEGF) [45]. Yang et al. [46] found miR-128b significantly up-regulated in plasma from ischemic stroke patients compared to healthy controls. In addition, miR-124 has previously been associated with ischemic stroke. Liu et al. [47] as well as Sun et al. [48] found an increase of miR-124 in the ischemic penumbra after permanent middle cerebral artery occlusion in mice. In both studies cell culture experiments indicated that miR-124 is involved in apoptosis. Recently, Li et al. [13] reported up-regulation of three miRNAs in serum of stroke patients including miR-1246, which had the highest fold change (FC = 13.6; *p* = 0.01) in our NGS experiment. In summary, these results, although not fully comparable, support our findings. Only one study besides ours has focused on miRNAs in CSF of stroke patients. Peng et al. [49] analyzed the expression of let-7e and miR-338 in CSF from ischemic stroke patients and found up-regulation of let-7e in a group of patients included 1–7 days after symptom onset (*n* = 11) but they did not include any brain-specific miRNAs in their analysis.

Despite our consistent experimental setup, uniform sample handling, and data processing we were not able to validate the results from our previous pilot study [27]. This might be due to the small differences found in our pilot study, or the fact that none of our pilot study *p* values passed a BH correction for multiple testing. These results highlight the importance of performing follow-up studies, especially when patient numbers are small. Exiqon’s protocol resulted in many missing values and had a lower detection rate for many miRNA sequences compared to NGS, which might explain why any potential differences in brain-enriched miRNAs were not detected in our previous pilot study. In addition, poorly elucidated factors like heterogeneity of genetic background, differences in BBB function, CSF turnover, and miRNA degradation might have influenced results differently in the two studies.

Limitations of our study include the relatively small study population and heterogeneous control group with regard to diagnoses. The various diagnoses represented in our control group may have influenced the fold changes calculated between stroke patients and controls. Nevertheless, if a stroke biomarker should be useful in the clinic, it should possess enough power to predict the diagnosis of ischemic stroke even when tested against non-healthy individuals. Therefore, and because of ethical issues in relation to doing lumbar punctures on healthy individuals, we decided to use neurological patients as controls.

We initially planned to include three equal groups of stroke patients with small-artery occlusion, large-artery atherosclerosis, and cardioembolism. However, we had to exclude patients from lumbar puncture if they were unable to give informed consent, received anticoagulant treatment, or had large infarctions with emerging vasogenic edema which contraindicated a lumbar puncture. Therefore, our study population mainly consisted of patients with lacunar infarctions and a smaller group of patients with larger infarcts having either cardioembolism or large-artery atherosclerosis, which prevented us from comparing miRNA expression profiles in subgroups based on stroke etiologies alone.

An advantage of our study is that it takes into consideration the technical uncertainties related to miRNA profiling by using three different profiling techniques on the same sample set. We observed quite significant discrepancies between results from NGS, qPCR (Exiqon), and qPCR (Applied Biosystems). The low quantities of miRNAs in the CSF combined with multiple factors such as RNA extraction quality, affinity of the primers, and PCR efficiency could influence the results in unpredictable ways. In addition, unknown biological factors such as BBB function and CSF clearance may have influenced our results.

Although the strongest miRNA expressional changes were seen in patients with larger infarcts, we attempted to analyze the predictive power of combining one or more miRNAs to separate stroke patients from controls with logistic lasso regression based on data from NGS. The best model combined 7 miRNAs but resulted in an overall unsatisfactory classification accuracy of 0.75.

## Conclusion

In conclusion, several brain-enriched miRNAs are elevated in the CSF three days after stroke onset, suggesting that these miRNAs reflect the brain damage caused by ischemia. The expressional differences seem, however, limited to patients with larger ischemic injury, which argues against the use of CSF miRNAs as diagnostic biomarkers of stroke based on current methods.

## Additional files

Additional file 1: Diagnosis, stroke subtype, infarct volume, and NIHSS of each patient included. (DOCX 20 kb)

Additional file 2: Comparison between study populations in our previous and present study. (DOCX 17 kb)

Additional file 3: Full description of the different protocols used for miRNA. (DOCX 31 kb)

Additional file 4: Venn diagram of miRNAs detected by NGS and qPCR extraction and miRNA profiling. (DOCX 192 kb)

## Abbreviations

AUC: Area under the curve
BBB: Blood brain barrier
BH: Benjamini-Hochberg
CML: Conditional maximum likelihood
CNS: Central nervous system
CSF: Cerebrospinal fluid
Ct value: Cycle threshold value
CT: Computed tomography
FDR: False discovery rate
HIF-1: Hypoxia inducible factor 1
IFC chip: Integrated fluidic circuit chip
KEGG: Kyoto Encyclopaedia of Genes and Genomes
miRNAs: MicroRNAs
MRI: Magnetic resonance imaging
NGS: Next Generation Sequencing
qPCR: Quantitative Polymerase Chain Reaction
REL: Relative expression value
ROC: Receiver Operating Characteristics
TMM: Trimmed Mean of M-values
TNF: Tumor necrosis factor
TOAST: Trial of Org 10,172 in Acute Stroke Treatment
VEGF: Vascular endothelial growth factor
