# Supplementary material for: Elevation of brain-enriched miRNAs in cerebrospinal fluid of patients with acute ischemic stroke
Source: Biomark Res. 2017 Jul 11;5:24. doi: 10.1186/s40364-017-0104-9 (PMC5504978; doi:10.1186/s40364-017-0104-9)
Supplement: Supplementary file 1 — Diagnosis, stroke subtype, infarct volume, and NIHSS of each patient included. (DOCX 20 kb) [file 40364_2017_104_MOESM1_ESM.docx]

**Additional file 1:** Diagnosis, stroke subtype, infarct volume, and NIHSS of each patient included

| Stroke group (n = 21) | | | | Control group (n = 21) | |
| --- | --- | --- | --- | --- | --- |
| Sex/Age | Diagnosis | Infarct (cm^3^) | NIHSS | Sex/Age | Diagnosis |
| F/77 | LAA | 2.5 | 0 | F/53 | Bells paresis |
| F/50 | LAA | 31.1 | 1 | M/31 | Lumbago |
| M/58 | LAA | 9.2 | 5 | M/64 | Lumbago |
| M/53 | LAA | 11.5 | 1 | M/47 | Polyneuropathy |
| F/76 | LAA | 32.4 | 2 | M/73 | Polyneuropathy |
| F/75 | CE | 30.0 | 4 | M/77 | Polyneuropathy |
| F/70 | CE | 6.6 | 3 | F/53 | Restless legs syndrome |
| M/47 | SAO | 0.3 | 2 | F/49 | Diplopia |
| M/65 | SAO | 1.2 | 3 | M/77 | Healthy |
| F/67 | SAO | 0.5 | 1 | F/69 | Depression |
| F/67 | SAO | 0.4 | 0 | F/32 | Multiple sclerosis |
| M/77 | SAO | 0.3 | 2 | F/70 | Amyotrophic lateral sclerosis |
| F/75 | SAO | 0.5 | 1 | M/86 | Mild cognitive impairment |
| M/58 | SAO | 1.1 | 10 | M/66 | Mild cognitive impairment |
| M/58 | SAO | 1.6 | 2 | M/59 | Toxic encephalopathy |
| F/75 | SAO | 1.3 | 4 | M/77 | Frontotemporal dementia |
| M/70 | SAO | 1.0 | 1 | M/64 | Dementia with Lewy bodies |
| M/68 | SAO | 0.6 | 1 | M/83 | Alzheimer’s disease |
| M/59 | O | 0.5 | 3 | M/73 | Alzheimer’s disease |
| M/78 | U | 0.3 | 3 | F/78 | Alzheimer’s disease |
| M/76 | U | 8.5 | 4 | M/85 | Alzheimer’s disease |

*NIHSS*: National Institute of Health Stroke Scale; *M*: male; *F*: female; *LAA*: stroke caused by large-artery atherosclerosis; *CE*: stroke caused by cardioembolism; *SAO*: stroke caused by small-artery occlusion; *O*: stroke of other cause (thrombophilia); *U*: stroke of undetermined etiology (all according to TAOST classification).
