# Supplementary material for: Elevation of brain-enriched miRNAs in cerebrospinal fluid of patients with acute ischemic stroke
Source: Biomark Res. 2017 Jul 11;5:24. doi: 10.1186/s40364-017-0104-9 (PMC5504978; doi:10.1186/s40364-017-0104-9)
Supplement: Supplementary file 2 — Comparison between study populations in our previous and present study. (DOCX 17 kb) [file 40364_2017_104_MOESM2_ESM.docx]

**Additional file 2:** Comparison between study populations in our previous pilot study and present follow-up study

| Variable | Pilot study (n=20) | Follow-up study (n=42) | Diff. (CI) | *P* value |
| --- | --- | --- | --- | --- |
| Mean age (total) | 65.10 | 66.31 | -1.2 (-9.6-7.1) | 0.71 |
| Mean age (stroke) | 73.40 | 66.62 | 3.3 (-0.7-13.6) | 0.05 |
| Mean age (control) | 56.80 | 66.00 | -6.6 (-23.5-5.1) | 0.19 |
| NIHSS (stroke) | 4.40 | 2.52 | 1.1 (-0.4-4.1) | 0.10 |
| SSS (stroke) | 48.40 | 51.76 | -2.5 (-8.8-2.1) | 0.21 |
| % males (total) | 0.60 | 0.60 | - | 1.00 |
| % males (stroke) | 0.50 | 0.57 | - | 0.50 |
| % males (control) | 0.70 | 0.62 | - | 1.00 |
| % stroke (total) | 0.50 | 0.50 | - | 1.00 |
| % SAO (stroke) | 0.30 | 0.52 | - | 0.22 |
| % LAA or CE (stroke) | 0.50 | 0.33 | - | 0.45 |
| % dementia (control) | 0.50 | 0.43 | - | 1.00 |
| % MS (control) | 0.30 | 0.05 | - | 0.08 |
| % healthy (control) | 0.20 | 0.43 | - | 0.26 |

*NIHSS*: National Institute of Health Stroke Scale; *SAO*: stroke caused by small-artery occlusion; *LAA*: stroke caused by large-artery atherosclerosis; *CE*: stroke caused by cardioembolism (all according to TAOST classification); *MS*: multiple sclerosis; *healthy*: patients with no evidence of CNS disease (e.g. polyneuropathy, benign headache or lower back pain). *P* values of differences in age and NIHSS are calculated with two-sided parametric T Test. *P* values of proportions are calculated with Fischer’s Exact Test.
