# Supplementary material for: Elevation of brain-enriched miRNAs in cerebrospinal fluid of patients with acute ischemic stroke
Source: Biomark Res. 2017 Jul 11;5:24. doi: 10.1186/s40364-017-0104-9 (PMC5504978; doi:10.1186/s40364-017-0104-9)
Supplement: Supplementary file 3 — Full description of the different protocols used for miRNA. (DOCX 31 kb) [file 40364_2017_104_MOESM3_ESM.docx]

#### Additional file 3: Full description of the different protocols used for miRNA extraction and miRNA profiling

#### Trizol RNA extraction followed by Illumina Next Generation Sequencing

RNA was isolated from 100 µl CSF using TriZol reagent (Invitrogen). CSF was thawed on ice and an aliquot of 100 µl were transferred to another tube containing 900 µl TriZol. The tube was mixed by pipetting and incubated for 10 minutes at room temperature followed by addition of 300 µl chloroform. The tube was shaken vigorously, incubated two minutes at room temperature and centrifuged at 12000 x g for 15 min. at 4 °C. The upper phase (containing RNA) was transferred to a new tube and 500 µl isopropanol and 1 µl glycogen (20 µg/µl) was added. RNA was precipitated overnight at -20 °C. The tubes were centrifuged at 16000 x g for 30 min. The supernatant was removed and the pellet was washed once with 85 % ethanol. The RNA pellet was dissolved in 10 µl water.

Small RNA libraries were prepared using the TruSeq small RNA library preparation kit (Illumina). The following changes to the protocol were applied: Ligation adapters were diluted 10 times. A total of 11 µl of the ligation product were used for reverse transcription. The RT primer were diluted 2 times and reverse transcription was performed in 20 µl instead of 10 µl. The resulting cDNA was amplified by 24 cycles of PCR in a 50 µl reaction volume. Amplified libraries were purified on 3% metaphor gels, quantitated using Picogreen and sequenced on a NextSeq500 (Illumina). The libraries from two stroke patient samples were of low quality and were therefore excluded from further analysis.

Reads were trimmed for adapters using Cutadapt^1^ and reads shorter than 15 nucleotides in length were discarded. Unique reads were counted using python scripts. In order to obtain expression values for miRNAs and other genes, read-counts were analyzed using sRNAbench^2^ using hg19 as reference and otherwise default settings. miRNA counts were normalized in R Bioconductor package edgeR^3^ using Trimmed Mean of M (TMM). Fold changes were based on the average count in each patient group.

#### Exiqon RNA extraction protocol and miRNA qPCR assays

RNA extractions and sample preparations were done by Exiqon Services, Denmark. Total RNA was extracted from 200 μl CSF using spin column chromatography (miRCURY^TM^ RNA isolation kit for bio fluids). RNA spike-in controls were added to the panel (Sp2, Sp4, and Sp5).

10 μl RNA was reverse transcribed in 50 μl reactions using the miRCURY LNA™ Universal RT miRNA PCR, Polyadenylation and cDNA synthesis kit (Exiqon). cDNA was diluted 50 times and assayed in 10 μl PCR reactions. Each miRNA was assayed once by qPCR on the miRNA Ready-to-Use PCR, Human panel I containing 372 specific miRNA primers. A DNA (Sp6) spike-in control was added to the panel. Negative controls were performed and profiled like the samples. Amplification was performed in a LightCycler® 480 Real-Time PCR System (Roche) in 384 well plates. Amplification curves were analysed using the Roche LC software, both for determination of Ct values and for melting curve analysis. The amplification efficiency was calculated by using LinReg software. All assays were inspected for distinct melting curves and the Tm was checked to be within known specifications for the assay. Both RNA and DNA spike-in control showed steady levels across all samples.

Only miRNAs detected with Ct < 37 and, in addition, 3 Ct values less than the negative control were included in the data analysis. Using NormFinder (ref) the best normalizer was identified as the average Ct value of the 9 miRNAs detected in all samples (miR-15a-5p, miR-21-5p, miR-23a-3p, miR-23b-3p, miR-99a-5p, miR-125b-5p, miR-145-5p, miR-204-5p, and miR-320a) using the formula $REL= {2^{miR average-miR}}/{2^{Global mean average-Global mean}}$ ^4^. Fold changes were based on the median value of REL in each patient group (missing values excluded).

#### Norgen Biotek RNA extraction protocol and Applied Biosystems qPCR assays

Total RNA was extracted from 200 μl CSF using spin column chromatography (Total RNA Purification Kit, Norgen Biotek) according to the protocol but with the following modifications: 10 mmol/L dithiothreitol (DTT) and 1.7 pmol/L synthetic cel-miR-54 were added to the lysis buffer. One µL of RNAse inhibitor (20 U/μl) was added to every elution tube before elution of RNA. Purified RNA samples were kept at -20 °C until used.

Reverse transcription was performed by using the TaqMan microRNA Reverse Transcription Kit (Applied Biosystems) with following modifications: The RT-primer-mix contained equal volumes of 24 different miRNA-specific stem-loop primers. The volume of each RT reaction was 10 µl including 1 µl Multiscribe reverse transcriptase, 1 µl 10x buffer reagent, 0.2 µl 100 mM dNTPs, 0.15 µl RNase inhibitor, 3 µl RT-primer-mix and 4.65 µl RNA purified from CSF. Reverse transcription was performed on a 2720 Thermal Cycler (Applied Biosystems). cDNA was kept at -20 °C until used. miRNA specific pre-amplification was accomplished according to the protocol for TaqMan PreAmp master mix and TaqMan MicroRNA Assays (Applied Biosystems) consisting of equal volumes of 48 assays (24 miRNA duplicates). cDNA was pre-amplified by 16 cycles of PCR performed on a 2720 Thermal Cycler (Applied Biosystems) and diluted 1:5 with H_2_O. Pre-amplified samples and TaqMan miRNA assays for 24 miRNAs (run in duplicates) were applied to a 48.48 Dynamic Array IFC chip for gene expression (Fluidigm) using loading reagents according to the manufacturer. A no template control (NTC) was included on the chip. Amplification with 40 PCR cycles was performed in a BioMark real-time PCR system (Fluidigm) using single probe settings (FAM-MGB, reference: ROX). Data was processed with Fluidigm PCR analysis software (v. 4.1.3) using auto detector settings. The RNA spike-in control (cel-miR-54) showed steady levels across all samples

Based on its steady level in our previous CSF study we had pre-selected miR-23a-3p as normalizer. However, miR-23a-3p was not stably expressed in this experiment, which made it unsuitable as a reference miRNA. Instead we used Normfinder^5^ which identified two equally good normalizers: miR-320a and the average Ct value of the 20 miRNAs detected in all samples (global mean). Based on the assumption that most of the 24 miRNAs in this validation experiment were differentially expressed between our patient groups we wanted to avoid a global mean normalization that could potentially undermine any differences by comparing raw data to the average of all miRNAs. Therefore, miR-320a was chosen as normalizer and relative expression levels were calculated using the formula $REL= {2^{miR average-miR}}/{2^{miR-320a average-miR-320a}}$^4^. Fold changes were based on the median value of REL in each patient group (missing values excluded).

#### References

1. Martin M. Cutadapt removes adapter sequences from high-throughput sequencing reads. *EMBnet.journal*. 2011;17:10–12.

2. Rueda A, Barturen G, Lebron R, Gomez-Martin C, Alganza A, Oliver JL, et al. SRNAtoolbox: An integrated collection of small RNA research tools. *Nucleic Acids Res.* 2015;43:W467–W473.

3. Robinson MD, McCarthy DJ, Smyth GK. edgeR: A Bioconductor package for differential expression analysis of digital gene expression data. *Bioinformatics*. 2009;26:139–140.

4. Jolla L. Quantification strategies in real-time PCR. In: Bustin S, editor. A-Z of Quantitative PCR. International University Line (IUL); 2004. p. 87–112.

5. Andersen CL, Jensen JL, Ørntoft TF. Normalization of Real-Time Quantitative Reverse Transcription-PCR Data : A Model-Based Variance Estimation Approach to Identify Genes Suited for Normalization, Applied to Bladder and Colon Cancer Data Sets. *Cancer Res.* 2004;64:5245–5250.
