# Supplementary material for: Elevation of brain-enriched miRNAs in cerebrospinal fluid of patients with acute ischemic stroke
Source: Biomark Res. 2017 Jul 11;5:24. doi: 10.1186/s40364-017-0104-9 (PMC5504978; doi:10.1186/s40364-017-0104-9)
Supplement: Supplementary file 4 — Venn diagram of miRNAs detected by NGS and qPCR extraction and miRNA profiling. (DOCX 192 kb) [file 40364_2017_104_MOESM4_ESM.docx]

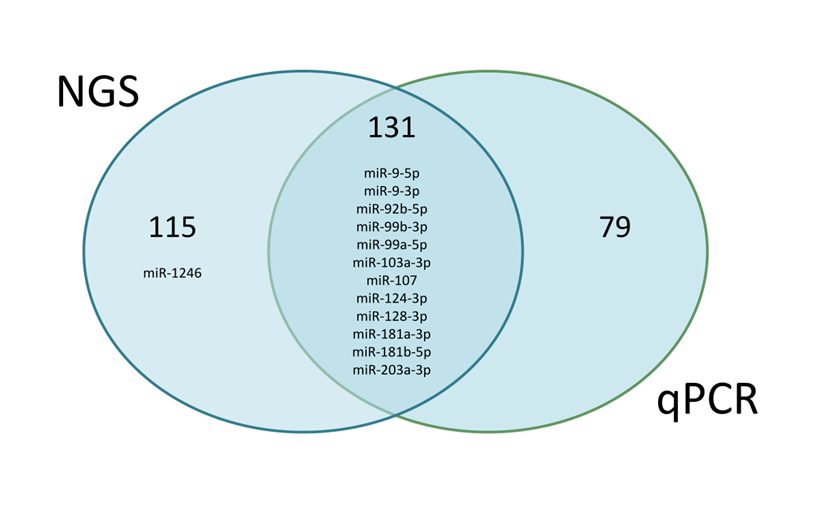


**Additional file 4:** Venn diagram showing numbers of miRNAs detected in CSF with Next Generation Sequencing and qPCR (human panel I, Exiqon) including lists of miRNAs that were differentially expressed in stroke patients in one or both experiments.
